# Natural Products: A Dependable Source of Therapeutic Alternatives for Inflammatory Bowel Disease through Regulation of Tight Junctions

**DOI:** 10.3390/molecules28176293

**Published:** 2023-08-28

**Authors:** Jing Peng, Hao Li, Oladejo Ayodele Olaolu, Saber Ibrahim, Sally Ibrahim, Shengyi Wang

**Affiliations:** 1 Key Laboratory of Veterinary Pharmaceutical Development of Ministry of Agriculture and Rural Affairs, Lanzhou Institute of Husbandry and Pharmaceutical Sciences of Chinese Academy of Agricultural Science, Lanzhou 730050, China; pj_lucky_7@163.com (J.P.); lihao_lh99@126.com (H.L.); aygenesis2succeed@yahoo.com (O.A.O.); 2 Department of Animal Health Technology, Oyo State College of Agriculture and Technology Igboora Nigeria, Igboora 201003, Nigeria; 3 Packaging Materials Department, National Research Centre, Giza 12111, Egypt; sa.ibrahim@nrc.sci.eg; 4 Nanomaterials Investigation Laboratory, Central Laboratory Network, National Research Centre, Giza 12111, Egypt; 5 Department of Animal Reproduction and AI, Veterinary Research Institute, National Research Centre, Dokki 12622, Egypt; sally_rashad2004@yahoo.com

**Keywords:** inflammatory bowel disease, natural products, intestinal barrier integrity, tight junction

## Abstract

Inflammatory bowel disease (IBD), which includes Crohn’s disease (CD) and ulcerative colitis (UC), can affect the entire gastrointestinal tract and mucosal layer and lead to intestinal damage and intestinal dysfunction. IBD is an inflammatory disease of the gastrointestinal tract that significantly impacts public health development. Monoclonal antibodies and other synthetic medications are currently used to treat IBD, but they are suspected of producing serious side effects and causing a number of other problems with long-term use. Numerous in vitro and in vivo studies have shown that organic macromolecules from plants and animals have an alleviating effect on IBD-related problems, and many of them are also capable of altering enzymatic function, reducing oxidative stress, and inhibiting the production of cytokines and release of proinflammatory transcriptional factors. Thus, in this paper, the natural products with potential anti-IBD activities and their mechanism of action were reviewed, with a focus on the protective effects of natural products on intestinal barrier integrity and the regulation of tight junction protein expression and remodeling. In conclusion, the insights provided in the present review will be useful for further exploration and development of natural products for the treatment of IBD.

## 1. Introduction

Inflammatory bowel disease (IBD), which includes Crohn’s disease (CD) and ulcerative colitis (UC), is a highly debilitating chronic inflammatory disease of the gastrointestinal tract which occurs at a peak age in adolescence and young adulthood and has a serious impact on public health. It can affect the entire gastrointestinal tract and mucosal layer, leading to intestinal damage and dysfunction. IBD patients commonly encounter symptoms such as abdominal pain, diarrhea, and rectal bleeding [1,2]. The specific pathogenesis of IBD is highly complex and remains unclear, and the likely involvement of genetic, environmental, and immunological factors has been reported [3]. In recent years, with changes in dietary patterns, the incidence and prevalence of IBD has increased rapidly in Asia and the Middle East and is expected to reach 1% by 2030 [4]. At present, there are no targeted drugs available for the treatment of IBD. Anti-inflammatory agents and immunosuppressive drugs, such as 5-amino salicylic acid (5-ASA) or adrenal cortex hormones, are mostly used in mild-to-moderate patients, but these drugs are either ineffective or have side effects including headache, nausea, vomiting, abdominal pain, and diarrhea [5,6]. For example, 5-ASA may induce sustained remission in only a small number of patients, whereas prolonged use of corticosteroids can trigger multiple side effects [7]. Biological therapies based on monoclonal antibodies have also been suggested for the treatment of IBD. However, their potential as therapeutic agents for IBD is severely constrained by the extremely high cost, parenteral administration, large proportion of non-responders, and immunologic problems [8,9]. Consequently, new approaches must be devised to treat IBD and restore intestinal mucosal homeostasis. Current studies on natural products suggest that single natural compounds, natural extracts, and natural compound preparations, especially natural single compounds extracted from daily foods and traditional Chinese medicine, have the potential to be used as drugs to alleviate intestinal inflammation in IBD.

## 2. The Pathogenesis of IBD

The pathophysiology of IBD has been better understood during the last few years, but its pathogenesis remains unknown. Studies of IBD pathogenesis have emphasized relevant disease mechanisms, including innate and adaptive immunity, as well as the interactions of genetic factors with microbial and environmental factors. Common clinical symptoms in patients with IBD include severe pain, abdominal pain, diarrhea, abscesses, and fistulas. The production of proinflammatory cytokines such as interleukin-1β (IL-1β), IL-6, IL-17, IL-22, IL-23, tumor necrosis factor-α (TNF-α), and interferon-α (IFN-α) highly shapes the development of IBD and results in excessive damage to intestinal tissues [10]. In addition, increased permeability of epithelial tight junctions (TJs) is a hallmark of intestinal inflammation in IBD, and disruption of the intestinal epithelial barrier structure and function is the most visible sign of IBD [11,12].

TJs are a multifunctional complex composed of multiple proteins. TJs are widely present at the top of the lateral membrane between the adjacent intestinal epithelial cells. At present, six complete transmembrane proteins have been identified, including occludin, claudins, JAM, CAR, trillion, angular, and two cytoplasmic proteins: ZO (ZO-1, -2, and -3) and cingulin [13]. Tight junctions between adjacent epithelial cells maintain high transepithelial electrical resistance by sealing the paracellular space between epithelial cells, controlling the flow of fluids and macromolecules between the blood and the intestinal lumen, and are primarily responsible for the structural integrity of the barrier (TEER) [14,15]. When the barrier is disrupted, potentially dangerous molecules from the intestinal lumen can disrupt the tight junction structure and increase intestinal permeability, which also disrupts immune homeostasis, increases gastrointestinal inflammation, and exacerbates intestinal disorders (e.g., IBD) [16].

**Figure 1:**
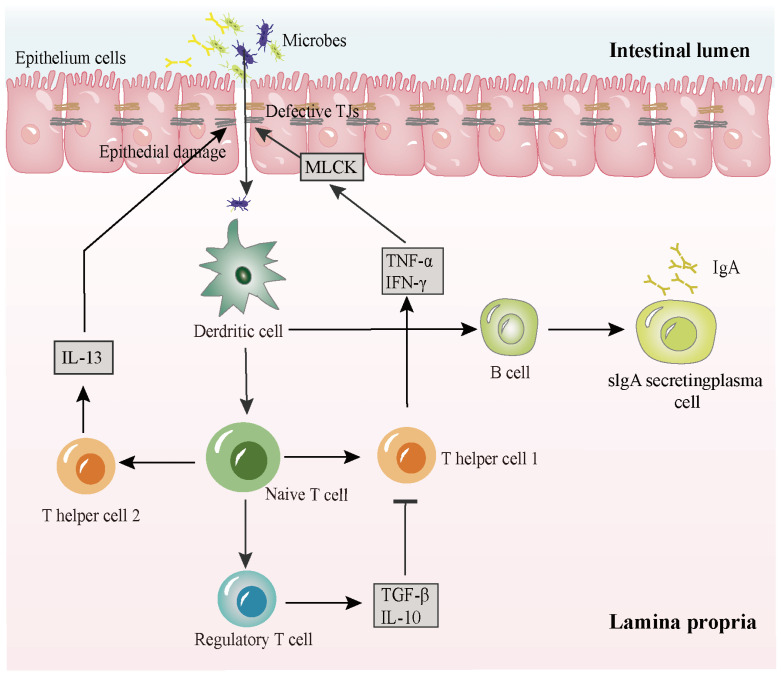
Cytokine network and tight junction (TJ) damage of inflammatory bowel disease. Macrophages and dendritic cells release inflammatory cytokines upon microbial stimulation. Stimulation of naïve T cells differentiates them into different subtypes, such as Th1 and Th2, which secrete IL-13, TNF-α, and IFN-γ; and induces B cells to produce secreted IgA (sIgA) antibodies; and regulatory T cells suppress inflammatory responses. Factors such as inflammatory factors and microorganisms can lead to the disruption of the structure of tight junction proteins, thereby undermining the integrity of the intestinal barrier. Thus, disruption of the balance of the intestinal immune system can lead to immune system dysregulation and the development of IBD.

Several pathological conditions can lead to decreased TJs and increased paracellular permeability of irregular TJs in IBD patients. The changes in the abundance and location of transmembrane TJ proteins such as ZO-1, claudin-1, and occludin are related to the increase in intestinal epithelial permeability and the development of gut disorders [17,18,19]. In contrast, a large number of in vivo and in vitro experiments have shown decreased expression of TJ-associated proteins and increased intestinal permeability due to reduced epithelial barrier function [20]. Impaired TJ function leads to intestinal antigen absorption and antigen translocation to the lamina propria, which triggers inflammatory response in the epithelium and immune cells (Figure 1) [21]. A relationship between the intestinal epithelial barrier and IBD has been reported, although the exact mechanism that triggers the disruption of gut homeostasis remains in question [22]. Moreover, maintaining the function and integrity of the intestinal epithelial barrier may contribute to the prevention and treatment of gut diseases, particularly IBD. Anatomical architecture protection of the intestinal luminal structure is essential to avert IBD and other related diseases.

## 3. Natural Products for the Treatment of IBD

Recently, a growing variety of natural products have been identified as immune nutrients that have immune-regulatory or anti-inflammatory effects on the human gut system. Different classes of natural products are mainly found in different plants, as shown in Table 1. Natural products in dietary and traditional Chinese medicine can influence the maintenance and enhancement of GI integrity through a variety of mechanisms, including alterations in signaling pathways associated with inflammation and expression of several tight junction proteins (claudins, occludin, and zonula occludens proteins), alterations in the expression of various cytokines, chemokines, complement components and their transcription factors, goblet cell abundance and mucin genes, and modulation of the cellular immune system. 

**Table 1:** Different classes of natural products are mainly found in different plants.

Classes of Single Bioactive Components	Monomers	Major Plants Present
Flavonoids	Kaempferol	Fruits, vegetables, and herbs [23]
	Quercetin	Flowers, leaves, and fruits of plant [24]
	Puerarin	*Pueraria lobata* [25]
	Naringin	Grapes and citrus fruits [26]
	Icariin	*Epimedium* [27]
	Alpinetin	*Alpinia katsumadai Hayata* [28]
	Baicalin	*Scutellaria baicalensis Georgi* [29]
	Rhein	*Rheum rhabarbarum* [30]
Terpenoids	Ginsenosides	*Ginseng* [31]
	Astragaloside IV	*Astragalus membranaceus* [32]
	Geniposide	*Gardenia jasminoides Ellis* [33]
	Patchouli alcohol	*P. cablin* [34]
	Atractylodes A	*Atractylodes macrocephala* [35]
	Clematichinenoside	*Clematis chinensis Osbeck* [36]
	Oridonin	*Rabdosia rubescens* [37]
Alkaloids	Berberine	*ranunculaceae, rutaceae and berberidaceae* [38]
	Koumine	*Gelsemium* [39]
Non-flavonoid polyphenols	Curcumin	*Turmeric (Zingiberaceae)* [40]
	Resveratrol	Red wine and grape skin [41]
Other classes	Emodin	*Rheum palmatum* [42]
	Arctigenin	*Fructus Arctii* [43]
	Sodium houttuyfonate	*Houttuynia cordata Thunb* [44]
	Artemisinin	*Artemisia annua* L. [45]

Several reports have indicated that natural phytochemicals, such as flavonoids, tannins, saponin, and phenolic compounds, have anti-inflammatory and antioxidant activities and can be used to treat IBD. For instance, when natural products such as thymoquinone, resveratrol, curcumin, and quercetin are encapsulated in nanoparticles, they have a great potential for the prophylaxis, management, and treatment of IBD [46]. The mechanism of action has been attributed to the regulation of various inflammatory mediators, such as IL-1β, IL-6, IL-10, TNF-α, prostaglandin E2 (PGE-2), inducible nitric oxide synthase (iNOS), and cyclooxygenase-2 (COX-2) [47,48]. In addition, natural macromolecules have been examined for their ability to inhibit the biochemical indices and molecular inflammatory pathways associated with IBD. Recent evidence has shown that natural products, such as flavonoids, alkaloids, terpenoids, and polyphenols, have therapeutic efficacy for IBD. The mechanism of action of these natural products is mostly or partially attributed to inhibiting the production of inflammatory factors, promoting the production of anti-inflammatory factors, and regulating the expression and distribution of tight junction proteins, thus protecting the integrity and function of the intestinal barrier. Therefore, the regulation of tight junctions by some natural products may be an alternative therapeutic tool for IBD. 

This review aims to provide an overview of the role of natural products from dietary and traditional Chinese medicine in anti-inflammatory, antioxidant, and tight junction modulation, as well as the potential application of dietary natural products in the treatment of IBD. Furthermore, we discuss the underlying mechanisms by which natural products exert their functions.

## 4. Flavonoids for the Treatment of IBD (Table 2)

### 4.1. Kaempferol

Kaempferol is a natural polyphenolic flavonoid antioxidant whose antioxidant and anti-inflammatory activities have been widely studied. Current studies have shown that kaempferol can regulate the expression and distribution of tight junctions by regulating NF-κB, MAPK/ERK, and PKA signaling pathways, thereby protecting the structural integrity and function of the intestinal barrier.

A study [49] demonstrated the mitigating effect of kaempferol on experimental colitis in mice. In the UC mouse model induced by sodium dextran sulfate (DSS), kaempferol exerted immunomodulatory effects in UC mice by regulating the gut microbiota and a variety of metabolites, improving the intestinal barrier, restoring the balance of gut microbiota, changing the metabolic profile, and inhibiting the TLR4-NF-κB signaling pathway. Pretreatment with kaempferol significantly relieves the symptoms of weight loss, diarrhea with mucus and blood, histological abnormalities, colon shortening, and abnormal intestinal permeability in UC model mice, in addition to dramatically alleviating DSS-induced intestinal barrier disruption by raising the levels of ZO-1, occludin, and claudin-1. Furthermore, kaempferol pretreatment decreased the levels of IL-1β, IL-6, and TNF-α, downregulated the transcription of a range of inflammatory signaling molecules, and increased IL-10 mRNA expression. 

Another study revealed that kaempferol also alleviated IL-8 secretion and barrier dysfunction of Caco-2 monolayer in an LPS-induced epithelial-endothelial co-culture model by inhibiting the activation of the NF-κB signaling pathway [50]. In a novel epithelial-endothelial cell co-culture model to investigate intestinal inflammation and barrier function, kaempferol was found to alleviate LPS-induced TEER reduction, FITC flux increase, IL-8 overexpression, and LPS-induced protein expression of NF-κB and phosphorylation of IκB and to ameliorate LPS-induced decreases of protein expression of ZO-1, occludin, and claudin-2, thereby protecting intestinal tight junctions and reducing intestinal mucosal barrier injury. 

Wang et al. [51], also found that kaempferol pretreatment could improve the integrity of Caco-2 cell monolayer barrier by affecting the expression and assembly of tight junction and adhesion connexin through the PKA pathway and the MAPK/ERK pathway. In this study, kaempferol pretreatment significantly ameliorated the impairment of the barrier function of Caco-2 monolayer by deoxyvaleryl alcohol and significantly increased the expression of claudin-3, claudin-4, occludin, and PKA, which favored the assembly of TJs. As a conclusion, kaempferol may offer a viable therapeutic option for IBD patients to reduce intestinal inflammation and its progression.

### 4.2. Quercetin

Quercetin is a widely distributed plant polyhydroxy flavonoid, found in flowers, leaves, and fruits of plants, which possesses a variety of biological activities and high medicinal value. It has been reported that quercetin has multiple pharmaceutical functions, including antioxidant, anti-inflammatory, anticancer, antibacterial, and anti-atherosclerotic properties [24]. Current studies have shown that quercetin can regulate the tight junction structure of intestinal epithelium and protect the integrity and function of the intestinal mucosal barrier by regulating Ca^2+^/JNK2/Src, TLR4/MyD88/p38MAPK, ERS, RhoA/ROCK, and Nrf2 signal pathways.

It has been reported that quercetin pretreatment of IEC-6 cells can resist indomethacin-induced barrier dysfunction by attenuating calcium-mediated JNK/Src activation [52]. In this study, indomethacin induction induced calcium-mediated JNK/Src activation in injured cells, resulting in barrier disruption. Quercetin pretreatment could significantly inhibit the activation of JNK/Src and increase the mRNA and protein expression of zonula occluden-1, occludin, and claudin-1 and alleviate intestinal barrier damage. Another study found that quercetin could improve the intestinal mucosal barrier by inhibiting the RhoA/ROCK signaling pathway and enhancing the mRNA and protein expression of tight junction proteins ZO-1, occludin, and claudin-1 in rat intestinal epithelial cells (IEC-6) [53]. In addition, in a rat model of acute necrotizing pancreatitis (ANP), quercetin inhibited the disruption of the intestinal barrier and the occurrence of inflammation by inhibiting TLR4/MyD88/p38MAPK and ERK [54]. In this study, quercetin reduced the overexpression of the proinflammatory factors IL-1β, tumor necrosis factors-α, and IL-17A, decreased plasma diamine oxidase (DAO), D-lactate, and endotoxin, and promoted the expression of intestinal tight junction proteins (including ZO-1, claudin-1, and occludin), which protects the integrity of tight junctions and improves the intestinal mucosal barrier. In in vitro studies, quercetin prevented diquat-induced cell death and intestinal barrier failure by activating Nrf2 in enterocytes [55]. Notably, quercetin regulates intracellular redox states via modulating tight junctions (ZO-1, ZO-2, ZO-3, occludin, and claudin-4) and increasing the quantity of antiapoptotic proteins in an Nrf2-dependent way. These findings suggest that quercetin was a potential candidate for the prevention and treatment of inflammatory bowel disease.

### 4.3. Puerarin

The root of *Pueraria lobata* (Gegen in Chinese) has long been used as traditional medicine. Puerarin is one of the main isoflavonoid components of the root of *Pueraria lobate* and its pharmacological properties have been widely investigated. Puerarin has been reported to be successfully utilized in the treatment of allergy disorders, neuronal protective diseases, and liver injury. Additionally, it has antioxidant, anti-inflammatory and antitumor properties [25]. The current study suggests that puerarin can protect the integrity of tight junction structures in the gut mucosa by regulating NF-κB signaling, MAPK (ERK1 and ERK2 signaling), and Nrf2.

Puerarin has been reported to promote the activation of the MAPK (ERK1, ERK2) signaling pathway and inhibit the activation of the NF-κB signal pathway related to TJ permeability and connectivity. In ethanol-induced Caco-2 monolayer, ethanol inhibited the expression of tight junction proteins (ZO-1, occludin, claudin-1) and increased the permeability of Caco-2 monolayer in Caco-2 cells by regulating the expression of NF-κB, MLCK, ERK1, and ERK2, thereby suppressing the expression and distribution of tight junction proteins (ZO-1, occludin, claudin-1), but the above effects induced by ethanol could be significantly antagonized by puerarin. A recent in vivo study found that puerarin modulates the intestinal epithelial barrier by inhibiting NF-κB and activating the Nrf2 signaling pathway, thus demonstrating a distinct protective effect against DSS-induced colitis in mice [55]. In this study, puerarin reduced myeloperoxidase (MPO) activity, colon shortening, and pathological damage to the colon and greatly reduced inflammation by downregulating NF-κB and inhibiting the release of proinflammatory mediators (TNF-α, IL-1β, and IL-6) in the body. Moreover, puerarin demonstrated antioxidative effects through controlling the expression of antioxidant enzymes (HO-1, NQO1, CAT, GSH, and SOD) and the Nrf2 pathway. Puerarin increases the expression of tight junction proteins (ZO-1, occludin, and claudin-1), thereby preventing the failure of the intestinal epithelial barrier. As a result, puerarin has the potential to be used as a natural treatment for inflammatory colitis diseases.

### 4.4. Naringin

Naringin is a well-known flavanone glycoside of grapes and citrus fruits that has been shown to have anti-inflammatory, anticancer and cardiovascular protective properties in vivo and in vitro. Naringin has been demonstrated to prevent experimental intestinal tumors and UC in mice [56]. The current study found that naringin can protect intestinal mucosal barrier function through either RhoA/ROCK/MLCK/MLC signaling pathways or RhoA/ROCK/NF-κB/MLCK/MLC signaling pathways in vivo or in vitro. 

Studies have shown that naringin improved sepsis-induced intestinal injury via RhoA/ROCK/NF-kB/MLCK/MLC signaling pathways in vivo and in vitro [47]. In this study, naringin significantly increased the survival rate of cecal ligation and puncture (CLP) mice, alleviated intestinal mucosal injury caused by sepsis, inhibited the release of TNF-α and IL-6 inflammatory factors, increased the level of anti-inflammatory factor IL-10, and promoted the expression of tight junction proteins ZO-1 and claudin-1 in vivo and in vitro through the RhoA/ROCK/NF-κB/MLCK/MLC signaling pathway. Furthermore, in another study, naringin was found to protect the integrity of the monolayer barrier of rat small intestine microvascular endothelial cells (RIMVECs) and to protect RIMVEC from TNF-α-induced apoptosis and migration inhibition [57]. This conclusion further supports naringin as a potential therapeutic agent for intestinal diseases.

### 4.5. Icariin

Icariin (ICA) is a representative flavonoid natural product, extracted from epimedium, that possesses a variety of biological activities and pharmacological effects including anti-inflammatory and antioxidant properties [58]. The phosphorylated derivative of ICA (pICA) has also been shown to possess potent anti-inflammatory and antioxidant properties. It has been reported that ICA has a protective effect against the disruption of intestinal barrier function in vivo and in vitro by inhibiting the expression of p38MAPK.

Previous studies have shown that ICA significantly attenuates LPS-induced intestinal damage, indicating that ICA may have a remarkable protective impact on the intestinal epithelium [59]. More importantly, ICA regulates the redox balance of intestinal epithelial cells by inhibiting the expression of p38MAPK, and thus, ICA has a protective effect against the disruption of intestinal barrier function. In addition, ICA pretreatment was shown to significantly increase the gene expressions of ZO-1, occludin, and claudin-1 in jejunal samples. Another study illustrated that ICA and its phosphorylated derivatives mitigated enterotoxin E. coli-induced disruption of the intestinal epithelial barrier by regulating p38MAPK [60]. This study showed that ICA and pICA regulated inflammatory response and oxidative stress in intestinal epithelial cells by inhibiting the expression of p38MAPK, thereby reducing the disruption of intestinal barrier function and abnormal intestinal permeability caused by ETEC (endotoxin *E. coli*) K88. These reports suggest that ICA and pICA may be effective natural products for preventing intestinal injury.

### 4.6. Alpinetin

Alpinetin, a major flavonoid isolated from *Alpinia katsumadai Hayata*, has been demonstrated to possess anti-colitis and intestinal barrier protective effects. Due to its antibacterial, antitumor, antioxidant, and anti-inflammatory bioactivities, alpinetin is being increasingly used in various medical applications [61]. Researches have shown that alpinetin protects the integrity and permeability of the intestinal epithelial barrier by regulating the Nrf2/HO-1 signaling pathway and AhR/SUV39H1/TSC2/mTORC1/ autophagy pathway.

Studies have shown that alpinetin can protect the integrity and permeability of intestinal epithelial barrier by regulating the expression of TJ proteins, and the possible mechanism of action is related to the inhibition of oxidative stress and activation of the Nrf2/HO-1 signaling pathway by alpinetin in DSS-induced UC mice [62]. Alpinetin has been reported to improve intestinal barrier function by improving DAI, colon shortening, histological score, and myeloperoxidase activity, inhibiting MDA levels, enhancing superoxide dismutase levels, upregulating occludin and ZO-1 expression, and downregulating claudin-2 expression. Another study found that alpinetin could improve the dynamic balance of the intestinal barrier by regulating AhR/SUV39H1/TSC2/mTORC1/autophagy pathways [63]. In addition, it has been reported that alpinetin significantly promotes intestinal barrier function by inhibiting the apoptosis of IECs, thus effectively treating colitis in mice. The specific mechanisms can be summarized as follows: activation of AhR, promotion of SUV39H1 degradation, increase in TSC2 expression, and inhibition of the mTORC1-mediated autophagy pathway. In conclusion, alpinetin showed excellent protective effect against intestinal barrier injury through ameliorating oxidative stress, promoting expression of tight junction protein, and inhibiting apoptosis, and is expected to be a brand-new natural medication for the treatment of UC.

### 4.7. Baicalin

*Scutellaria baicalensis Georgi* is a medicinal plant with a long history of use in the treatment of diseases including hypertension and intestinal disorders. It contains a significant amount of the flavone compound baicalin, which has antibacterial, anti-inflammatory, antivirus, antioxidant, and anticancer properties and is used in the treatment of inflammatory bowel diseases [64]. This study suggests that baicalin may protect the integrity of the intestinal mucosal barrier and its normal function by regulating signaling pathways associated with STAT1 and STAT3.

Recent studies have found that baicalin has a protective effect on intestinal integrity under the condition of hypertension [65]. Baicalin treatment reduced the hypertension-related elevation of intestinal permeability and decreased the levels of inflammatory indexes such as SHR high sensitivity C-reactive protein, IL-1β, and IL-6 in a model of intestinal injury induced by spontaneously hypertensive rats (SHRs). Additionally, baicalin significantly protected intestinal integrity in SHRs by protecting intestinal ultrastructure and increasing gene expression of intestinal tight junction proteins (such as ZO-1 and occludin). Moreover, baicalin treatment enhanced the abundance of SCFA-producing bacteria in SHRs and the number of SCFAs in feces. Another study found that baicalin can reduce proinflammatory cytokines, such as IL-2, IL-6, IL-8, IL-1, and SIgA, in mouse models of RV-DH diarrhea, reverse RV-DH diarrhea in mice, and significantly improve lung and intestinal histological damage [66]. In this study, baicalin treatment reversed the change in the ratio of CD4+ and CD8+ cells in intestinal mucus, indicating that baicalin may control the immune response in the lung and intestinal mucosa. Mechanistically, baicalin may alleviate RV-DH diarrhea via modulating STAT1- and STAT3-related signaling pathways as well as by increasing the expression of occludin, claudin-1, and ZO-1. These studies point to baicalin possessing good therapeutic and immunoregulatory abilities that may be directly applied to the treatment of IBD.

### 4.8. Rhein

Rhein is a major flavonoid isolated from *Rheum rhabarbarum* that has been shown to have antioxidant and anti-inflammatory effects in previous studies [67]. For thousands of years, enteritis, gastritis, edema, and other illnesses have been successfully treated with *rheum rhabarbarum* in traditional Chinese medicine. Current studies have found that rhein regulates tight junctions by regulating NF-κB/MLCK/p-MLC, NLRP3, MAPKs (p38MAPK and JNK), and Nrf2 pathways, thereby protecting intestinal barrier function. 

Rhein has been found to restore intestinal barrier function by inhibiting the NF-κB/MLCK/p-MLC pathway [68]. In an LPS-stimulated IEC-6 cell model, rhein restored the expression and distribution of ZO-1, alleviated the increase in phenol red flux and the decrease in TEER, and weakened MLC phosphorylation, MLCK expression, and NF-κB activation. Meanwhile, rhein decreased LPS-stimulated IL-1β and IL-6 secretion levels, downregulated the expression of TLR4 and NLRP3, cleaved caspase1, and inhibited the expression of NF-κB. Mechanistically, rhein restored the intestinal epithelial barrier by inhibiting the NF-κB/MLCK/p-MLC pathway and inhibited LPS-induced IL-1β and IL-6 expression by modulating the TLR4/NF-κB pathway and NLRP3 inflammasome.

In addition, it has been reported that rhein may protect the intestinal barrier from LPS-induced oxidative and inflammatory damage through inhibition of p38 MAPK and JNK as well as activation of the Nrf2 pathway [69]. In a rat model stimulated by intraperitoneal injection of LPS, pretreatment with rhein dramatically increased intestinal ZO-1 and occludin levels. Rhein also inhibited LPS-induced intestinal inflammation and oxidative stress by decreasing serum and intestinal levels of TNF-α, IL-1β, IL-6, and NO, increasing intestinal catalase and glutathione peroxidase (GSH-Px) activities, upregulating HO-1 expression, and downregulating intestinal malondialdehyde (MDA) levels. From the perspective of mechanism, rhein exerts its anti-inflammatory and antioxidative effects by suppressing p38 MAPK and JNK and activating the Nrf2 pathway, thereby alleviating LPS-induced intestinal barrier injury. In summary, rhein has a certain therapeutic effect on intestinal inflammatory diseases by alleviating the intestinal inflammatory response, oxidative stress, and the expression of tight junction proteins.

**Table 2:** The effects and mechanism of action of flavonoids on gut barrier.

Monomers	Objects (Model Induces)	Effects	Signaling Pathway
Kaempferol	1. A model of colitis mice induced by dextran sulfate sodium 2. Epithelial-endothelial cells co-culture model 3. Caco-2 cell caused by Deoxynivalenol	1. ZO-1, occludin, and claudin-1 ↑, IL-1β, IL-6, and TNF-a ↓, IL-10 ↑ 2. TEER ↑, FITC ↓, ZO-1, occludin, and claudin-2 ↑, NF-κB and I-κB ↓ 3. Claudin-3, claudin-4, and occludin ↑, PKA ↑	1. Inhibiting the LPS-TLR4-NF-κB pathway [49] 2. Inhibiting NF-κB signaling pathway activation [50] 3. Activating the PKA pathway and deactivation of the MAPK/ERK pathway [51]
Quercetin	1. IEC-6 cell injured by indomethacin 2. A rat intestinal epithelial (IEC-6) cells 3. In a rat model of acute necrotizing pancreatitis (ANP) 4. IPEC-1 incubated with vehicle or diquat	1. ZO-1, occludin, and claudin-1 ↑ 2. ZO-1, occludin, and claudin-1 ↑ 3. ZO-1, claudin-1, occludin ↑, IL-1β, TNF-α, and IL-17A ↓ 4. ROS ↓, GSH ↑, ZO-1, ZO-2, ZO-3, occludin, and claudin-4 ↑	1. Attenuating calcium-mediated JNK/Src activation [52] 2. Inhibiting the RhoA/ROCK signal pathway [53] 3. Inhibiting TLR4/MyD88/p38MAPK and ERS [54] 4. Activating Nrf2 [55]
Puerarin	1. Ethanol-induced Caco-2 monolayer 2. DSS-induced colitis mice	1. ZO-1, occludin, claudin-1 ↑, NF-κB ↓, MLCK, ERK1 and ERK2 ↑ 2. TNF-α, IL-1β, IL-6 ↓, Nrf2, HO-1, and NQO1 ↑, MDA ↓, CAT, GSH, and SOD ↑, ZO-1, occludin, and claudin-1 ↑	1. Activation of the MAPK (ERK1 and ERK2) signal pathway and inhibition of the NF-κB signal pathway [35] 2. Inhibition of NF-κB and activation of the Nrf2 signaling pathway [25]
Naringin	1. CLP mice and lipopolysaccharide (LPS)-stimulated MODE-K cells	1. TNF-α and IL-6 ↓, IL-10 ↑, ZO-1, and claudin-1 ↑, p65 and IκB-α ↓, P-MLC and MLCK ↓, GTP-RhoA ↓	1. Inhibiting the RhoA/ROCK/NF-kappaB/MLCK/MLC signaling pathway [47]
Icariin	1. Bisphenol A(BPA)-exposed mice and MODE-K cells 2. Piglets and IPEC-J2 cell with ETEC K88	1. ZO-1, occludin, and claudin-1 ↑, ROS, RNS, MDA, andH_2_O_2_ ↓, SOD, GPx, CAT, and T-AOC) ↑ 2. ZO-1 and occludin ↑, IL-1β, IL-6, IL-8, and TNF-α ↓, ROS, MDA, and H_2_O_2_ ↓, p38 MAPK ↓	1. Inhibiting p38 MAPK [42] 2. Regulating the expression of p38 MAPK [60]
Alpinetin	1. A mouse model of (DSS)-induced ulcerative colitis 2. A mouse model of DSS-induced UC and in TNF-α-stimulated Caco-2 and NCM460 cells	1. DAI and SOD ↑, MDA ↓, occludin and ZO-1 ↑, claudin-2 ↓ 2. TEER ↑, claudin-7 and occludin ↑	1. Activation of the Nrf2/HO-1 signal pathway [62] 2. Regulating the AhR/SUV39H1/TSC2/mTORC1/ autophagy pathway [63]
Baicalin	1. A mouse model of pediatric RV-DH diarrhea	1. Occludin, claudin-1, and ZO-1↑, IL-1β, IL-2, IL-6, and IL-8 ↓, SIgA ↓	1. Inhibiting STAT1 and activating the STAT3 signaling pathways [66]
Rhein	1. An IEC-6 cell model with LPS stimulation 2. A rat model induced by intraperitoneal injection of lipopolysaccharide (LPS)	1. ZO-1 ↑, p-MLC, MLCK, NF-κB ↓, IL-1β, and IL-6 ↓, TLR4, NLRP3, and cleaved caspase1 ↓, NF-κB ↓ 2. DAO, ZO-1, and occludin ↑, TNF-α, IL-1β, IL-6, and NO ↓, CAT, GSH-Px, and HO-1 ↑, MDA ↓	1. Inhibition of the NF-κB/MLCK/p-MLC pathway, TLR4/NF-κB pathway, andNLRP3 inflammasome [68] 2. Inhibiting the MAPKs (p38MAPK and JNK) signaling pathways, activating Nrf2 pathway [69]

Notes: The ↑ indicates positive regulation, while the ↓ indicates negative regulation.

## 5. Terpenoids for the Treatment of IBD (Table 3)

### 5.1. Ginsenosides

Ginseng contains proto-ginsenosides Rg1 and Re, as well as protopanaxadiol-type ginsenosides Rb1 and Rb2 [70]. Traditional Chinese medicine has employed ginseng root as a tonic medication for more than 2000 years. Ginsenosides are the molecules that confer pharmacological activity to ginseng, with Rg1 being the most abundant [71]. Ginsenosides exhibit a wide range of pharmacological effects including immunomodulatory, antitumor, and anti-inflammatory effects. The current study found that ginsenosides maintain the structural integrity and normal function of the intestinal mucosa by regulating the TLR4/NLRP12/NF-κB signaling pathway and the NLRP3 inflammasome pathway, thereby protecting against intestinal inflammatory injury.

Studies have shown that ginsenoside Rg1 markedly reduces the release of proinflammatory cytokines (IL-1β and TNF-α) and greatly reduced the inflammatory response in dendritic cells of DSS-induced UC mice. The mechanism may act by interfering with the inflammation-dereived signaling pathway of TLR4/NLRP12-/NF-κB [72]. In another study, ginsenoside Rk3 was found to ameliorate DSS-induced UC by protecting intestinal barrier function and inhibiting NLRP3 inflammasome expression [73]. In this study, Rk3 decreased the expression of proinflammatory factors (TNF-α, IL-1β, and IL-6), NLRP3, ASC, and Caspase-1 and increased the levels of TJ (ZO-1, occludin, and claudin-1), and SCFA, which suggests that blockade of the NLRP3 inflammasome pathway attenuates colonic inflammation and protects the intestinal epithelial barrier. Therefore, inhibiting the activation of the NLRP3 inflammasome may be the reason why the inflammatory response is weakened. In addition, ginsenoside Rb1 was shown to protect the intestinal mucosal barrier from harm by peritoneal air exposure, which may be associated with its anti-inflammatory effects [74]. In conclusion, ginsenosides can effectively regulate the expression of tight junctions to protect against intestinal barrier injury.

### 5.2. Astragaloside IV

*Astragalus membranaceus* has been used in traditional Chinese herbal medicine for at least 2000 years. Astragaloside IV (AS-IV), a tiny saponin, is one of the main active components extracted from *Astragalus membranaceus* root [75]. The results of the current study showed that AS-IV reduced intestinal barrier damage and TJ disruption by inhibiting the activation of RhoA/NLRP3 inflammasome signaling in both an in vivo model of CLP-induced sepsis and an in vitro model of LPS-induced Caco-2 monolayer barrier [76]. In CLP-induced sepsis mice, AS-IV decreased mortality, cytokines release, IFABP secretion, intestinal histological scores, and barrier permeability, and increased expression of tight junction proteins (occludin and ZO-1). Additionally, 200 g/mL AS-IV co-incubation decreased cytokine levels in LPS-induced Caco-2 cells and improved gut barrier function in vitro without cytotoxicity. These findings provide a feasible mechanistic explanation for the therapeutic effects of AS-IV in sepsis.

### 5.3. Geniposide

Geniposide is an iridoid glycoside extracted from the fruits of *Gardenia jasminoides Ellis*, which possesses anti-inflammatory, antioxidative, and antitumor effects [77]. Studies have shown that geniposide can improve barrier dysfunction by inhibiting the AMPK-mediated MLCK pathway.

The results of the current investigation showed that geniposide improved TNBS-induced experimental colitis in vivo and attenuated LPS-induced barrier dysfunction in vitro by decreasing the production of proinflammatory cytokine and improving impaired intestinal barrier function [78]. Geniposide injection significantly reversed weight loss and improved the condition of colitis and related symptoms in rats with TNBS-induced colitis. Additionally, geniposide reduced colonic neutrophil infiltration and the release of inflammatory cytokines (TNF-α, IL-1β, and IL-6). In an in vitro model, geniposide improved LPS-induced endothelial barrier failure in Caco-2 cells by dose-dependent TER enhancement. The results of in vivo and in vitro experiments showed that geniposide upregulated the expression of tight junction proteins (occludin and ZO-1), promoted AMPK phosphorylation, and downregulated the expression of NF-κB, COX-2, iNOS, and MLCK. Moreover, it has been demonstrated that AMPK overexpression and AMPK siRNA transfection reversed the low expression of MLCK protein expression induced by geniposide, indicating that geniposide improves barrier dysfunction through AMPK-mediated suppression of the MLCK pathway. Thus, geniposide ameliorated TNBS-induced experimental rat colitis by decreasing inflammation as well as modifying compromised epithelial barrier function through activation of the AMPK signaling pathway. In conclusion, these results imply that geniposide may have clinical application value for the treatment of inflammatory bowel disease.

### 5.4. Patchouli Alcohol

Patchouli alcohol (PA), a tricyclic sesquiterpene component extracted from *P. cablin*, is an active compound with anti-gastro ulcerogenic, anti-IBD, and strong gastrointestinal protective effects. PA has now been found to protect intestinal barrier function by inhibiting the TLR2/MyD88/NF-κB pathway.

Studies have shown that PA has a therapeutic effect on rat intestinal mucositis established by intraperitoneal injection of 5-fluorouracil (5-FU), and the possible mechanisms involve reducing inflammation, protecting the intestinal mucosal barrier, and improving intestinal microflora [79]. By preventing IκBα phosphorylation and p65 translocation from the cytoplasm to the nucleus, PA could successfully block the production of TLR2 and MyD88 and inactivate the NF-κB pathway, which increased the levels of the anti-inflammatory cytokine IL-10 and decreased the levels of the proinflammatory cytokines (TNF-α, IL-1β, IL-6, and MPO). In addition, PA enhanced the expression of MLC, ZO-1, occludin, claudin-1, and mucin-2 proteins, decreased the relative mRNA level of MLCK, and protected the intestinal mucosal barrier. PA has been also reported to restore microbial diversity and rebalance microbial communities, particularly by increasing the relative abundance of *Bifidobacteria* and *Lactobacilli* while decreasing the relative abundance of *Bacteroides*, *Helicobacter*, and *Parabacteroides*. Therefore, PA may prevent the onset and progression of intestinal mucositis by reducing inflammation, protecting the mucosal barrier, and controlling intestinal microbiota.

### 5.5. Atractylodes A

Atractylodes A (AA) is a guaiacol-type sesquiterpene glycoside isolated from *Atractylodes macrocephala* with protective effects on the intestinal mucosal barrier. It was found that AA could improve gastrointestinal function by regulating the p38MAPK signaling pathway. 

Previous studies have found that extract of *Atractylodes macrocephala* protects the intestinal mucosal barrier in rats with splenic deficiency syndrome by inhibiting the p38MAPK signaling pathway, increasing the expression of tight junction proteins (ZO-1 and occludin), and inhibiting p38MAPK phosphorylation [80]. Another study demonstrated that AA protected the intestinal mucosal barrier by inhibiting the p38 MAPK pathway in a rat model of spleen deficiency syndrome [81]. This study noted that AA enhanced the levels of MTL, GAS, c-Kit, intestinal propulsion, body weight, and gastric residual volume while reducing the proliferation of cellular nuclear antigens, epidermal growth factor receptor levels, and intestinal tissue damage. Validation studies showed that AA treatment increased the expression of ZO-1 and occludin in tight junctions and reduced the phosphorylation of p38MAPK and MLC. However, even though AA may be an effective new drug in IBD treatment, further in vivo and clinical data are necessary to confirm its role.

### 5.6. Atractylodes Clematichinenoside

Clematichinenoside AR (AR) is a triterpenoid saponin extracted from the roots of *Clematis chinensis Osbeck* and is the main active ingredient of the plant. AR has been shown to be very effective in reducing inflammation and controlling immune response, which helps to avoid experimental rheumatoid arthritis [82]. It has now been found that AR can protect the structural integrity and function of the intestinal barrier by regulating tight junctions through inhibition of the PI3K/Akt signaling pathway.

Researchers have found that AR can significantly reduce blood–brain barrier (BBB) damage by lowering the levels of TNF-α and IL-1β and increasing the expression of TJ proteins including ZO-1 and occludin [83]. In addition, AR improves gastrointestinal function by suppressing the intestinal mucosal immune system and PI3K/Akt signaling pathway [84]. Reductions in inflammation scores, disease activity index (DAI), and levels of inflammatory markers in Il-10/mice indicated that AR treatment dramatically alleviated spontaneous colitis. It was also reported that the effects of AR were related to the protection of intestinal barrier function (increased the expression of occludin and ZO-1) and the maintenance of immune system homeostasis (suppression of Th17 cell function through the promotion of Treg responses). Furthermore, AR treatment protected IL-10^−/−^ mice from injury by decreasing epithelial cell apoptosis. All of these findings suggest that AR has a strong protective effect against celiac-like colitis, and they provide a new therapeutic drug option for patients with celiac disease.

### 5.7. Oridonin

Oridonin is a diterpenoid molecule isolated from the bacterium *Rabdosia rubescens* which has been shown to have a number of pharmacological and physiological activities, including anti-inflammatory, antibacterial, and antitumor effects [85]. It has been illustrated that oridonin protects intestinal barrier function by regulating the PxR/NF-κB signaling pathway. Moreover, oridonin attenuates TNBS-induced post-inflammatory irritable bowel syndrome (PI-IBS) by regulating the PXR/NF-κB signaling pathway [86]. In a PI-IBS rat model and Caco-2 cell lines, oridonin significantly ameliorated the impaired barrier function and upregulated the expression of TJ (claudin-1, occludin, and ZO-1) proteins. Another study demonstrated that oridonin reduced inflammation by blocking the phosphorylation of NF-κB p65 and the expression of its downstream genes (iNOS, COX-2, IL-1β, and IL-6). The molecular mechanism may be related to the regulatory effect of oridonin on PXR/NF-κB. The above findings suggest that oridonin may be considered as an effective therapeutic drug for the treatment of PI-IBS.

**Table 3:** The effects and mechanism of action of terpenoids on gut barrier.

Monomers	Objects (Model Induces)	Effects	Signaling Pathway
Ginsenoside Rg1	A mouse model of colitis induced by sodium glucan sulfate (DSS)	IL-1β and TNF-α ↓	Interfering with TLR4-NLRP12-NF-κB [72]
Ginsenoside Rk3	A mouse model of colitis induced by DSS	TNF-α, IL-1β, IL-6, NLRP3, ASC, and Caspase-1 ↓, ZO-1, occludin, and claudin-1 ↑	Blockading of the NLRP3 inflammasome pathway [73]
Astragaloside IV	Septic mice modeled by cecal ligation and puncture (CLP) operation and LPS-challenged Caco-2 monolayer barrier model	Occludin and ZO-1 ↑, Caspase-1, IL-1β, and IL-18 ↓	Suppressing RhoA/NLRP3 inflammasome signaling [76]
Geniposide	Rats with TNBS-induced colitis and Caco-2 cells-induced LPS	TNF-α, IL-1β, and IL-6 ↓, NF-κB, COX-2, iNOS, and MLCK ↓, occludin and ZO-1 ↑, p-AMPK ↑	Activating the AMPK signaling pathway, inhibiting the MLCK pathway [78]
Patchouli alcohol	Rat intestinal mucositis model established by intraperitoneal injection of 5-fluorouracil (5-FU)	TLR2 and MyD88 ↓, NF-κB p-IκBα and p65 ↓, TNF-α, IL-1β, IL-6, and MPO ↓, IL-10 ↑, MLC, ZO-1, occludin, claudin-1, and mucin-2 ↑	Inhibiting the TLR2/MyD88/NF-κB pathway [79]
Atractylodes A	A rat model of spleen deficiency syndrome (SDS)	ZO-1 and occludin ↑, p-p38MAPK and p-MLC ↓	Inhibition of the p38 MAPK pathway [81]
Clematichinenoside AR	In a spontaneous colitis mice model by in interleukin-10 gene knockout (IL-10^−/−^)	Occludin and ZO-1 ↑, IL-17A+CD4+T cells Bcl-2, caspase-3, and Bax ↓	Inhibiting the PI3K/Akt signal pathway [84]
Oridonin	In a PI-IBS rat model and Caco-2 cell lines	Claudin-1, occludin, and ZO-1 ↑, p-NF-κB, and p65 ↓, iNOS, COX-2, IL-1β, and IL-6 ↓	Inhibiting PxR/NF-κB signaling [86]
Saikosaponin-d	Dextran sulfate sodium (DSS)-induced ulcerative colitis (UC) mice	TNF-α, IL-6, and IL-1β ↓, IL-10 ↑, Muc1 and Muc2 ↑, ZO-1 and Claudin-1 ↑	Inhibiting NF-κB activation [87]
Morroniside and loganin	DSS-induced murine model of colitis and an LPS-induced colorectal cancer (CRC) cell inflammation model	ZO-1, occludin, claudin-3, Ecadherin, and Muc2 ↑, IL-1β, IL-6, TNF-α, and IFN-γ ↓, p-STAT3 and p-p65 ↓	Blocking of the STAT3/NF-κB pathway [88]

Notes: The ↑ indicates positive regulation, while the ↓ indicates negative regulation.

## 6. Alkaloids for the Treatment of IBD (Table 4)

### 6.1. Berberine

Berberine is an alkaloid compound, extracted from herbs such as *Cortex phelloderm* and *Rhizoma coptidis*, which has anti-inflammatory properties. Berberine is considered by traditional Chinese medicine (TCM) to be useful in the treatment of gastroenteritis, abdominal pain, and/or diarrhea due to its low cost and side effects [89]. It has been shown that berberine protects the intestinal barrier by regulating TLRs/NF-κB, IGF-1/IGFBPs signaling, Wnt/β-catenin signaling, and NF-κB/MLCK signaling. 

A previous study has found that berberine alleviated DSS-induced colitis by improving intestinal barrier function and reducing inflammation and oxidative stress [90]. This study demonstrated that berberine attenuated weight loss, colon shortening, and colon damage in DSS-induced colitis mice, significantly inhibited the increase in fluorescein isothiocyanate-dextran in serum, and decreased ZO-1, occludin, and epithelial cadherin expression in colonic tissues. At the same time, berberine also significantly inhibited the expression of IL-1β, IL-6, and TNF-α mRNA and the phosphorylation of STAT3. Furthermore, in colon and serum samples, berberine decreased myeloperoxidase levels while increasing catalase and superoxide dismutase levels. In conclusion, this study suggests that berberine may alleviate colitis by promoting intestinal barrier function, inhibiting proinflammatory cytokine expression and oxidative stress response, and regulating the STAT3 signaling pathway.

In an IBS-D mouse model, berberine increased the production of tight junction proteins and repaired damage to the epithelial barrier by inhibiting the activation of the NF-B/MLCK pathway and reducing the expression of TNF-α [91]. In this study, berberine significantly elevated the levels of occludin, claudin-1, ZO-1, and F-actin in the intestinal epithelium of 4% acetic acid-induced IBS-D mice and significantly decreased the levels of TNF-α, NF-κB p65, MLCK, MLC, TRAF6, and RIP1, which exerted a protective effect on the intestinal barrier.

In a rat model of acute endotoxemia induced by LPS injection, berberine promotes the mRNA and protein expression levels of ileal insulin-like growth factor I (IGF-I) and binding protein 3 (IGFBP-3), increases the expression of ileal occludin and claudin-1, and thus repairs the LPS-induced disruptions of intestinal tight junctions and restores intestinal mucosal function [92]. In addition, inhibition of IGF-I/IGFBP-3 signaling by AG1024 or siRNAs reduced these protective effects of berberine. 

In another study, berberine was found to have a protective impact on GVB function in sepsis, and its effects were directly correlated with the modification of the Wnt/beta-catenin signaling pathway [93]. Berberine significantly decreased endotoxin levels and vascular leakage in a rat CLP sepsis model. And berberine had the same antagonistic effect on LPS-induced injury to rat intestinal microvascular endothelial cells (RIMECs), such that it resulted in a significant decrease in endothelial permeability of RIMECs to FITC-dextran, an elevation of TEER, and an increase in the expression of claudin-12, β-catenin, and VE-cadherin. 

Oxyberberine (OBB), an intestinal microflora metabolite of berberine, has recently been reported to significantly alleviate DSS-induced experimental colitis by preserving colonic integrity, suppressing inflammatory responses, and modifying gut microflora characteristics [94]. In DSS-induced colitis mice, OBB dramatically reduced DSS-induced dysbacteriosis and restored the dysbacteriosis to normal levels. OBB also increased the expression of TJ proteins such as ZO-1, ZO-2, JAM-A, claudin-1, and occludin, which exerted a protective effect against DSS-induced disruption of the intestinal epithelial barrier. In addition, OBB significantly reduced the levels of colonic inflammatory cytokines by downregulating the protein expression of TLR4 and MyD88, inhibiting the phosphorylation of IκBα and the migration of NF-κB p65 from the cytoplasm to the nucleus, and blocking the TLR4/MyD88/NF-κB signaling pathway. Collectively, these results suggest that berberine may be a new and promising natural product for the treatment of IBD.

### 6.2. Koumine

Koumine is a potent antioxidant and an abundant alkaloid monomer in the genus *Gelsemium*. Due to its anti-inflammatory, antioxidative, analgesic, and growth-promoting properties, koumine has attracted much attention in the animal feed and breeding industries [95]. Previous study has suggested that koumine may, to some extent, have a protective effect against H_2_O_2_-induced apoptosis. In IPEC-J2 cells exposed to H_2_O_2_, koumine was cytoprotective by reducing ROS generation, inhibiting caspase-3 activity, and modulating the expression of Bax and Bcl-2. Another study found that koumine prevented intestinal barrier dysfunction and attenuated endotoxin-induced oxidative stress and inflammation by regulating the NRF2/NF-κB signaling pathway [96]. In a model of LPS-induced intestinal barrier dysfunction in IPEC-J2 cells, koumine treatment attenuated LPS-induced intestinal barrier dysfunction but had no significant effect on tight junction proteins ZO-1, claudin-1, and occludin. Furthermore, koumine significantly inhibited LPS-induced inflammatory response and downregulated the levels of inflammatory factors, including TNF-α, IL-6, IL-1β, NO, iNOS, and COX-2, that were closely related to its inhibition of the NF-κB pathway, the reduction of IκBα and NF-κB phosphorylation, and the reduction of p-p65 nuclear translocation. Koumine also reduced LPS-induced increase in ROS and MDA content by activating the Nrf2 pathway, increased the degradation level of Nrf2 and HO-1 by KEAP-1, and promoted the production of antioxidant enzymes such as SOD and CAT. The above studies strongly suggest that the protective impact of koumine on intestinal inflammatory disease injury may be due to its ability to reduce intestinal oxidative stress and inflammation, thereby confirming koumine as a potential therapeutic option for intestinal inflammatory diseases.

**Table 4:** The effects and mechanism of action of alkaloid on gut barrier.

Monomers	Objects (Model Induces)	Effects	Signaling Pathway
Berberine	1. A model of colitis mice induced with DSS 2. In a mice model of IBS-D established by using 4% acetic acid 3. A rat model of acute endotoxemia induced by injection of lipopolysaccharide (LPS) 4. DSS-induced colitis mice	1. ZO-1, occludin, and epithelial cadherin ↑, IL-1β, IL-6, and TNF-α ↓, P-STAT3 ↓, MPO ↓, and SOD, CAT ↑ 2. Occludin, claudin-1, ZO-1, and F-actin ↑, TNF-α, NF-kB p65, MLCK, MLC, TRAF6, and RIP1 ↓ 3. Ileal insulin-like growth factor I (IGF-I) and binding protein 3 (IGFBP-3) ↑, occludin and claudin-1 ↑ 4. ZO-1, ZO-2, JAM-A, claudin-1, and occludin ↑, TLR4 and MyD88 ↓, P-IκBα and NF-κB p65 ↓	1. Inhibiting the STAT3 signaling pathway [90] 2. Inhibiting the activation of the NF-κB-MLCK pathway [91] 3. Modulation of the Wnt/beta-catenin signaling pathway [93] 4. Blocking the TLR4-MyD88-NF-κB signaling pathway [94]
Koumine	1. IPEC-J2 cells induced by lipopolysaccharide	1. TNF-α, IL-6, IL-1β, NO, iNOS, and COX-2 ↓, p-IκBα and NF-κB p-p65 ↓, Nrf2 and HO-1 by KEAP-1 ↑, SOD and CAT ↑	1. Inhibition of the NF-κB pathway, activating the Nrf2 pathway [96]

Notes: The ↑ indicates positive regulation, while the ↓ indicates negative regulation.

## 7. Non-Flavonoid Polyphenols for the Treatment of IBD (Table 5)

### 7.1. Curcumin

Curcumin is a polyphenolic compound present in the turmeric (Zingiberaceae) plant. Curcumin exhibits a variety of bioactivities including protection of cellular homeostasis, reduction of endoplasmic reticulum stress and oxidative stress, and anti-inflammatory potential [97]. Recent research has revealed that curcumin can regulate tight junctions and intestinal barrier function by regulating MAPK, AMPK- TFEB, and NF-κB signaling pathways. 

It has been reported that curcumin pretreatment dramatically reduced the levels of the master cytokine IL-1 secreted by LPS-induced IECs and macrophages [98]. Curcumin also inhibited IL-1β-induced activation of p38 MAPK in IECs, leading to elevated MLCK expression, phosphorylation of tight junction proteins (ZO-1, claudin-1, and claudin-7), and disruption of the normal arrangement of actin filaments. The primary site of action of curcumin is anticipated to be the intestinal barrier and IECs. Despite its low bioavailability, curcumin may affect chronic inflammatory disorders by lowering intestinal barrier dysfunction.

Another study found that curcumin promotes Parkin-dependent mitophagy via the AMPK-TFEB signaling pathway to reduce oxidative stress-induced intestinal barrier injury and mitochondrial damage [99]. In this study, curcumin was found to effectively attenuate oxidative stress, intestinal epithelial barrier damage, and mitochondrial damage caused by hydrogen peroxide (H_2_O_2_) in porcine intestinal epithelial cells (IPEC-J2 cells) in a mode of action that was dependent on PTEN-induced putative kinase (PINK1)-Parkin mitophagy. Mechanistically, curcumin improved the intestinal barrier and mitochondrial function in H_2_O_2_-induced IPEC-J2 cells by abolishing the mitophagy-related protein Parkin. Notably, the antioxidant properties of curcumin were reduced in H_2_O_2_-treated IPEC-J2 cells when PRKAA1 was depleted. Furthermore, curcumin significantly increased the nuclear translocation and transcriptional activity of transcription factor EB (TFEB), which was alleviated by cotreatment with compound C, an AMPK inhibitor. This indicates that curcumin stimulates TFEB transcription via the AMPK signaling pathway. The addition of curcumin to the diet of a porcine model of diquat-induced oxidative stress improved its redox status, reduced mitochondrial damage, induced mitophagy, and influenced the AMPK-TFEB signaling pathway. These findings suggest that curcumin triggers Parkin-dependent mitophagy through the activation of AMPK and subsequent nuclear translocation of TFEB, thereby alleviating oxidative stress, improving intestinal barrier function, and strengthening mitochondrial function.

In addition, nanoparticle curcumin prevented the development of DSS-induced colitis by inhibiting NF-κB activation and increasing the number of Tregs [100], which was accompanied by changes in the fecal SCFA levels and gut bacteria composition. Nanoparticle curcumin showed significant therapeutic effects on weight loss, disease activity index, histological colitis score, and mucosa permeability. According to immunoblotting analysis, curcumin nanoparticle therapy dramatically reduced NF-κB activity in colonic epithelial cells and mRNA expression of inflammatory mediators in the mucosa. Curcumin nanoparticle therapy enhanced the number of butyrate-producing bacteria and the quantity of fecal butyrate and resulted in more frequent expansion of CD103+CD8α-regulatory dendritic cells and CD4+ Foxp3+ regulatory T cells in the colonic mucosa. These findings demonstrate that curcumin offers a novel therapeutic possibility for the treatment of IBD, despite its poor absorption and limited bioavailability.

### 7.2. Resveratrol

Resveratrol (3,4′,5-trihydroxy-trans-stilbene; RES) is a polyphenolic acid that is abundant in red wine and grape skins. In recent years, RES has been shown to have a variety of other health-promoting effects in addition to beneficial effects on the coronary arteries, neurological, hepatic, and cardiovascular systems. It has been demonstrated that RES can inhibit the development of a number of cancer cells, as well as oxidative stress, viral infection, inflammation, and platelet aggregation. Recent studies have found that RES protects the integrity and function of the intestinal mucosal barrier by regulating MAPK signaling molecules and the PI3K/Akt-mediated Nrf2 signaling pathway.

It has been illustrated that RES attenuates oxidative stress-induced intestinal barrier injury through the PI3K/Akt-mediated Nrf2 signaling pathway [101]. By inhibiting the DON-induced decrease in transepithelial electrical resistance and increase in paracellular permeability, RES was able to prevent DON-induced bacterial translocation. Mechanistic research proved that RES defended against DON-induced barrier dysfunction by encouraging claudin-4 assembly in the tight junction complex, which was likely mediated by regulating the secretion of IL-6 and IL-8 through mitogen-activated protein kinase-dependent pathways. Furthermore, this study demonstrated that RES significantly decreased DON-induced phosphorylation of p38, ERK, and JNK, indicating that MAPK signaling molecules are involved in the regulation of TJ structure and function.

Another study found that RES activates the Nrf2 signaling pathway through PI3K/Akt to reduce the damage of oxidative stress on the intestinal barrier [102]. This study evaluated the effects of RES on H_2_O_2_-induced oxidative stress in IPEC-J2 cells and found that RES pretreatment significantly improved the activities of SOD1, CAT, and GSH-Px, expression levels of tight junction proteins (claudin-1, occludin, and ZO-1), and decreased H_2_O_2_-induced intracellular ROS levels and apoptosis. Additionally, under conditions of oxidative stress, RES increased the phosphorylation of Akt, Nrf2, and the expression levels of the antioxidant genes HO-1, SOD-1, and CAT in a dose-dependent manner. Short-hairpin RNA (shRNA)-mediated Nrf2 knockdown reduced RES-induced upregulation of TJ protein levels and antioxidant gene expression as well as RES-mediated protection against H_2_O_2_-induced apoptosis. Consistent with Nrf2 knockdown, the PI3K/Akt inhibitor LY294002 significantly reduced the effects of RES on Nrf2 phosphorylation, TJ protein levels, and antioxidant gene expression under oxidative stress conditions. These results suggest that RES can directly protect IPEC-J2 cells from oxidative stress and DON-induced intestinal damage via the Nrf2 signaling pathway, indicating that RES may be a useful feed additive for the prevention of intestinal damage in livestock production.

**Table 5:** The effects and mechanism of action of non-flavonoid polyphenols on gut barrier.

Monomers	Objects (Model Induces)	Effects	Signaling Pathway
Curcumin	1. The human IEC lines Caco-2 and HT-29 induced with LPS 2. H_2_O_2_ induced oxidative stress in IPEC-J2 cell and in a piglet’s intestinal oxidative stress model by challenging with diquat 3. BALB/c mice were fed with 3% DSS	1. MLCK ↓, IL-10 ↑, and IL-1β ↓, p38MAPK ↓, ZO-1, claudin-1, claudin-7, and actin filaments ↑ 2. SOD, CAT, Cu/Zn-SOD, Mn-SOD, GPX-1, and GPX-4 ↑, MDA and ROS ↓, FD4 flux ↓, TER ↑, occludin, ZO-1, and claudin-1, PINK-1 and Parkin ↑ 3. CD4+ Foxp3+regulatory T cells and CD103+CD8α ↑, TNF-α, IL-1β, IL-6, CXCL1, and CXCL2 ↓	1. Inhibition p38MAPK [98] 2. Activation of the AMPK-TFEB signal pathway [99] 3. Suppressing NF-κB [100]
Resveratrol	1. IPEC-J2 cell induced by Deoxynivalenol 2. Oxidative stress induced by H_2_O_2_ in IPEC-J2 cells	1. TEER ↑, promoting the assembly of claudin-4, IL-6, and IL-8 ↓, reduced DON-induced phosphorylation of p38, ERK, and JNK 2. claudin-1, occludin, and ZO-1 ↑, superoxide dismutase-1 SOD-1, CAT, and GSH-Px ↑, ROS and apoptosis ↓, p-Akt, p-Nrf2, and HO-1, SOD-1, and CAT ↑	1. Suppressing MAPK signaling [101] 2. Inhibiting the PI3K/Akt-mediated Nrf2 signaling pathway [102]

Notes: The ↑ indicates positive regulation, while the ↓ indicates negative regulation.

## 8. Other Classes of Single Bioactive Components from Herbs for the Treatment of IBD (Table 6)

### 8.1. Emodin

Emodin is an anthraquinone compound isolated from the traditional Chinese medicine *Rheum palmatum*, which has been demonstrated to have vasorelaxant, antitumor, antimicrobial, and anti-inflammatory properties. In China, *rheum palmatum* has a long history of use in the treatment of intestinal obstruction. Studies have shown that emodin acts as a laxative by controlling the contractility of intestinal smooth muscle, which in turn controls the movement and physiological function of the muscle [103]. At present, it has been found that emodin can protect against intestinal inflammatory injury and maintain the normal structure and function of the intestinal mucosa by regulating the NF-κB signaling pathway, RhoA/ROCK, and NOTCH signaling pathway.

Emodin has been indicated to protect the TJ barrier from damage by inhibiting the HIF-1α and NF-κB signaling pathways to mitigate LPS- and HR-induced intestinal epithelial barrier dysfunction (manifested by reduced expression of ZO-1) [104]. In LPS- and HR (hypoxia-reoxygenation)-treated Caco-2 monolayers, emodin significantly reduced elevated paracellular permeability and decreased transepithelial electrical resistance. Moreover, emodin decreased the expression of HIF-1α, IκB-α, NF-κB, and COX-2 in a dose-dependent manner and significantly attenuated the damage caused by LPS and HR (manifested by decreased expression of the TJ protein, ZO-1). Another study found that emodin may play an important role in attenuating this damage by regulating the intestine apoptotic pathway through controlling the activity of the RhoA/ROCK and NOTCH signaling pathways [105]. 

The experiment also demonstrated that emodin could regulate the RhoA/ROCK and NOTCH signaling pathway by modulating the expression of miR-218-5p, thus inhibiting the activity of the apoptosis-related proteins Fas, FasL, Bax, caspase-9, and caspase-3 in the intestine, increasing the expression of intestinal occludin, ZO-1, and E-cadherin, and decreasing the permeability of intestinal mucosa. Ultimately, emodin protected the intestinal mucosa from acute severe pancreatitis and attenuated the damage of the intestinal mucosal barrier. These results suggest that emodin, as a traditional therapeutic approach, may be crucial in preventing intestinal barrier damage caused by IBD.

### 8.2. Arctigenin

*Fructus Arctii* is a traditional herb medicine used in the treatment of inflammatory diseases with a wide range of biological activities including anti-inflammatory, anticancer, neuroprotective, antioxidant, and endoplasmic reticulum stress regulation. Arctigenin is the main active component of *Fructus arctii*, which has inhibitory effects on proinflammatory factors such as nuclear factor kappa B, inducible nitric oxide synthase, and oxidative stress, thereby supporting its anti-inflammatory and antioxidant properties [106]. 

Studies have reported that arctigenin promotes the expression of TJ proteins via the ERβ-MLCK/MLC pathway, thereby maintaining the integrity of the intestinal epithelial barrier to ameliorate IBD [107]. It has also been demonstrated that arctigenin maintains the intestinal epithelial barrier function in DSS- and TNBS-induced colitis mice by regulating the ERβ-MLCK/MLC pathway. In Caco-2 and HT-29 cells, arctigenin reduced TNF-α and IL-1β-induced changes in F-actin localization, enhanced TEER, restored aberrant tight junction protein expression, and decreased monolayer permeability. In addition, arctigenin has been shown to increase the expression of tight junction proteins through the ERβ-MLCK/MLC pathway to maintain the integrity of the intestinal epithelial barrier in IBD patients. Thus, arctigenin may be an effective natural drug for the treatment of IBD.

### 8.3. Sodium Houttuyfonate

Sodium houttuyfonate (SH) is a derivative of houttuyfonate, the active ingredient of *Houttuynia cordata Thunb*, which possesses anti-inflammatory effects and stabilizing properties. It has been reported that SH protects the intestinal barrier and reduces the generation of intestinal proinflammatory cytokines by controlling the NF-κB signaling pathway [108]. In a mouse model of diarrhea induced by *Salmonella typhimurium* (ST), SH treatment maintained the morphology of the jejunum mucosa and reduced the pathological damage of colon tissue. In addition, SH safeguarded the intestinal barrier by controlling the localization and distribution of tight junction proteins. Meanwhile, SH also markedly decreased the production of proinflammatory cytokines (TNF-α, IL-1β, and IL-6) as well as inflammation-related enzymes (iNOS and COX-2). Mechanistically, SH acts at the molecular level by blocking the phosphorylation of NF-κB p65 and the degradation of the IκB protein. In conclusion, the above work revealed the preventive effects on intestinal barrier damage and anti-inflammatory potential of SH and provided new perspectives on how SH protects the intestinal tract.

### 8.4. Artemisinin

Artemisinin is a type of sesquiterpene lactone drug derived from *Artemisia annua* L. with a peroxide bridge, which is commonly used to treat malaria caused by multidrug-resistant strains of Plasmodium falciparum [109]. A growing number of data suggest that the antimalarial medicine artemisinin (ART) and its derivatives have potent anti-inflammatory and immune-regulatory capabilities. Among them, artesunate (ARS), a water-soluble semi-synthetic derivative of ART, may treat experimental colitis by regulating the immune system [110]. 

Recent studies have found that ARS protects the integrity and function of the intestinal mucosal barrier by regulating the NF-κB signaling pathway [111]. ARS treatment significantly attenuated the clinical symptoms of DSS-induced ulcerative colitis in mice, including inhibition of body weight loss, reduction of disease activity index (DAI) score, and prevention of intestinal shortening. Additionally, ARS treatment significantly reduced DSS-induced erosion of surface epithelial cells, reduction of goblet cells, and destruction of the crypt along with inflammatory cell infiltration. In the presence of a high Bcl-2/Bax ratio, ARS significantly reduced the loss of Muc2 and claudin-1 in the mucosal layer and decreased the expression of cleaved-caspase-3 in colon tissues.

Furthermore, this study reconfirmed that ARS significantly inhibited the activation of the NF-κB signaling pathway, phosphorylation of IκBα and NF-κB p65, the expression of IL-1β, IL-6, and TNF-α, and augmented the expression of IL-10. These findings suggest a protective effect of ARS against ulcerative colitis (UC).

**Table 6:** The effects and mechanism of action of other natural products on gut barrier.

Monomers	Objects (Model Induces)	Effects	Signaling Pathway
Emodin	1. Caco-2 cells induced by LPS/hypoxia-reoxygenation 2. Rat intestinal epithelial cell-6, pancreatitis model rats induced by Taurocholate	1. ZO-1 ↑, HIF-1α, IκB-α, NF-κB, and COX-2 ↓ 2. Fas, FasL, Bax, caspase-9, and caspase-3 ↓, occludin, ZO-1, and E-cadherin ↑	1. Inhibiting the HIF-1α and NF-κB signaling pathways [104] 2. Regulate the activity of the RhoA/ROCK and NOTCH signaling pathways [105]
Arctigenin	Colitis mice induced by DSS, TNBS, (Caco-2 and HT-29 cell lines), TNF-α, and IL-1β	Occludin, ZO-1, and F-actin ↑, TEER ↑	Inhibiting the ERβ-MLCK/MLC pathway [107]
Sodium houttuyfonate (SH)	A mouse model of diarrhea induced by Salmonella typhimurium (ST)	TNF-α, IL-1β, and IL-6 ↓, iNOS, COX-2 ↓, p-NF-κBp65, and IκB ↓, the localization and distribution of tight junction proteins ↑	Inhibiting the NF-κB signaling pathway [108]
Artemisinin	A mouse model of ulcerative colitis induced by DSS	Muc2 and claudin-1 ↑, Bcl-2/Bax ↓, cleaved-caspase-3 ↓, p-IκBα and NF-κBp65 ↓, IL-1β, I L-6, and TNF-α ↓, IL-10 ↑	Inhibiting the NF-κB signaling pathway [111]

Notes: The ↑ indicates positive regulation, while the ↓ indicates negative regulation.

## 9. Conclusions

Due to the rising incidence of inflammatory bowel disorder (IBD) worldwide and the side effects and limitations of existing therapeutic agents, new therapeutic methods for IBD need to be developed urgently. Natural products are known to be a huge source for the development of novel drugs. A variety of natural components that are extracted from natural productions have received increasing attention from researchers because they have antioxidant effects and significant abilities to reduce intestinal inflammation. Numerous studies in the literature have demonstrated the antioxidant and anti-inflammatory properties of natural products used in traditional Chinese medicine.

**Figure 2:**
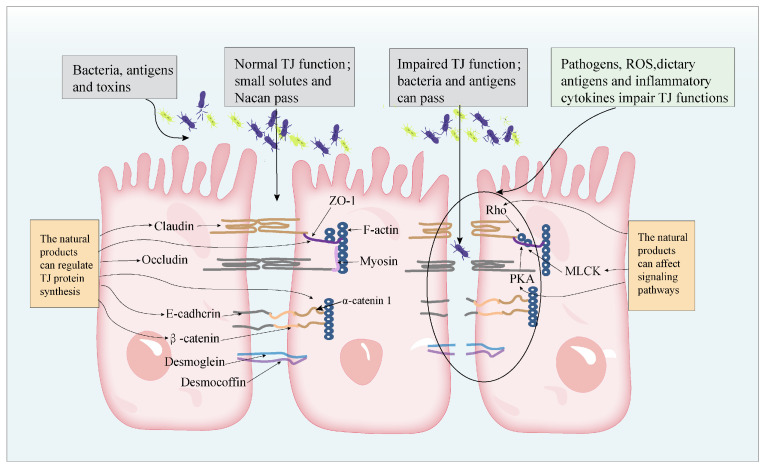
Schematic representation of the different mechanisms through which natural products protect the integrity of the gut. Pathogenic microorganisms, dietary antigens, reactive oxygen species (ROS) generated by different stressors, and inflammatory cytokines synthesized by immune homeostasis impair the barrier function of the tight junction (TJ) by altering the expression, localization, and modulation of signaling pathways of TJ proteins. Natural products can directly modulate the synthesis of TJ proteins or the signaling pathways of TJ regulatory proteins, leading to phosphorylation of TJ proteins and alteration of the surrounding functional actin ring.

**Figure 3:**
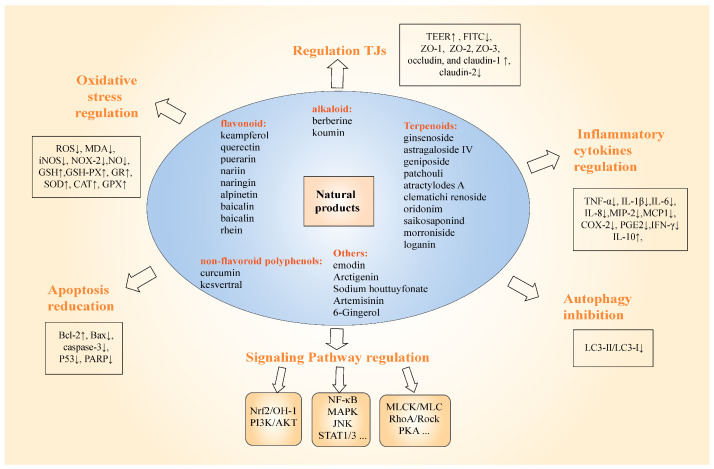
Emerging investigations have shown the anti-IBD effects of the bioactive ingredients from natural products. The ↑ indicates positive regulation, while the ↓ indicates negative regulation.

In this review, we summarize the therapeutic effects of natural products on IBD and related mechanistic studies. Natural products have been reported to have a variety of effects on the integrity of the gastrointestinal tract (GI) in patients with IBD, including effects on the alteration of signaling pathways, on the expression of several tight junction proteins (claudins, occludins, and zonula occludens proteins), on the expression of different cytokines, chemokines, complement components, and their transcription factors, on the abundance of goblet cell and the expression of mucin gene expression, and on the regulation of the cellular immune system. In addition, studies in the literature have reported that inflammatory factors play a strong role in regulating the expression and distribution of tight junctions. Therefore, as shown in Figure 2, the protective effects of natural products on tight junctions in the intestinal barrier are mainly reflected in two aspects: on the one hand, the effects of natural products on intestinal tight junctions are mainly through the direct regulation of the expression and distribution of tight junction proteins, and, on the other hand, natural products reduce the secretion of harmful cytokines through their anti-inflammatory and antioxidant functions to reduce cytokine damage to tight junctions. It is noteworthy that different classes of natural products have been found to have certain similarities in their mechanism of action in the treatment of inflammatory bowel disease, mainly including the regulation of the following signaling pathways. Among them, signaling pathways related to tight junctions mainly include: MLCK/MLC, RhoA/ROCK, and PKA signaling pathways (Figure 3). Signaling pathways related to anti-inflammation include NF-κB, MAPK (ERK, JNK, NF-κB, MAPK (ERK, JNK, p38MAPK), STAT1/3, NLRP3, and AMPK signaling pathways. Signaling pathways related to antioxidant properties include Nrf2, Nrf2/HO-1, and PI3K/Akt signaling pathways. Finally, apoptosis-related signaling pathways include Bcl-2/Bax, caspase-3 signaling pathways, etc. In addition, we also found that flavonoids and terpenoids have been extensively studied in the treatment of inflammatory bowel disease, and they can exert protective effects on tight junctions by modulating tight junction-related signaling pathways and anti-inflammatory and antioxidant-related signaling pathways. For example, quercetin was found to exert protective effects on tight junctions by regulating the JNK/Src signaling pathway [52], the RhoA/ROCK signaling pathway [53], TLR4/MyD88/p38MAPK and ERS [55], TLR4/MyD88/p38MAPK and p38MAPK, the p38MAPK and ERS signaling pathways [54], and Nrf2 [55].

However, current clinical studies on dose selection may not be sufficient to support better effects of natural products in IBD treatment. This is because doses too low may not be efficacious, while high doses may produce toxicity and impair barrier function. Therefore, dose selection of natural plant bioactive compounds (PBCs) is critical. Ideally, a higher dose in the GI tract will limit the growth of pathogenic microorganisms, but that dose may negatively affect GI functions. PBCs may strengthen the GI epithelial barrier, but they may also make it more difficult for nutrients to be absorbed from both the rumen and the intestine. Therefore, it is difficult to select an effective dose that allows specific PBCs to significantly inhibit the growth of pathogenic microorganisms while maintaining or improving GI tract functions. Notably, these findings are more often the result of in vitro experiments, and in vivo validation has only been carried out in mouse experiments; hence, it is necessary to elucidate more precisely the mechanisms of action and targets of natural products at the molecular level in order to have a better understanding of the protective effects of natural products on the intestinal barrier for their application in biotechnology and pharmaceuticals.

In addition, with the discovery of new mechanisms of action in recent years, the activities of natural products of dietary sources are broader and more diverse than initially recognized. The epigenetic effects of natural products are a significant example. The first study on the inhibition of DNA methyltransferases by dietary phytochemicals used catechins from tea [112]. Natural products are thought to play a role in controlling cellular epigenetic activity, which has been demonstrated in a range of tissues and organs, particularly in the intestinal epithelial cells [113]. Therefore, dietary natural products have received increasing attention as functional foods with anti-inflammatory effects [61].

This review enumerates and describes the anti-inflammatory properties of different natural compounds and their potential mechanisms in the prevention and treatment of IBD, as mentioned in previous studies. These studies have demonstrated that a variety of natural compounds can prevent or treat colitis by affecting the process of inflammatory cytokine production. Therefore, natural anti-inflammatory substances are preferred in the development of drugs for the treatment of colitis. However, the majority of experimental studies have used mice as models with different doses of the same compound to show its anti-inflammatory activity. There are still no conclusive clinical studies that indicate the ideal therapeutic dose for patients with colitis. Although the development of natural anti-inflammatory compounds for the treatment of IBD has great potential, more studies on their safety, dose screening, and metabolism are required. The research advancements and viewpoints presented in this review provide reliable and reproducible data that will benefit future research and development of natural products against IBD.

## Data Availability

All relevant data are within the paper.

## Sample Availability

Samples of the compounds are not available from the authors.
